# Impact of Blank Holding Force and Friction on Springback and Its Prediction of a Hat-Shaped Part Made of Dual-Phase Steel

**DOI:** 10.3390/ma16020811

**Published:** 2023-01-13

**Authors:** Peter Mulidrán, Emil Spišák, Miroslav Tomáš, Janka Majerníková, Jana Bidulská, Róbert Bidulský

**Affiliations:** 1 Institute of Technology and Materials Engineering, Faculty of Mechanical Engineering, Technical University of Košice, Mäsiarska 74, 040 01 Kosice, Slovakia; 2 Faculty of Materials, Metallurgy and Recycling, Technical University of Kosice, Park Komenskeho 10, 040 01 Kosice, Slovakia; 3 Bodva Industry and Innovation Cluster, Budulov 174, 045 01 Moldava nad Bodvou, Slovakia

**Keywords:** springback, dual-phase steel, simulation, friction, blank holding force

## Abstract

Formability and its prediction of high-strength steels is an important research subject for forming specialists and researchers in this field. Springback and its accurate prediction of high-strength steels are very common issues in metal forming processes. In this article, the impact of blank holding force and friction on the parts springback made of dual-phase steel was studied. Numerical predictions of the springback effect were conducted using nine combinations of yield criteria and hardening rules. Results from experiments were evaluated and compared with results from numerical simulations. The use of lower blank holding forces and PE foil can reduce springback by a significant amount. Numerical simulations where the Yoshida-Uemori hardening rule was applied produced more accurate springback prediction results compared to simulations that used Krupkowski and Hollomon’s isotropic hardening rules in number of cases.

## 1. Introduction

Springback is usually defined as a negative phenomenon that occurs after the forming operation, external force removal. It changes the dimensions and geometry of the formed part, which has an undesirable impact on the part accuracy and can lead to assembly problems [1,2,3]. Especially in the automotive industry, springback can lead to production errors, where the car body mostly consists of a large amount of sheet metal pressings with different mechanical properties which are joined together by welding, clinching, glueing, etc., using programmed manufacturing robots [4,5,6]. Steels with higher values of yield strength display more springback compared to mild steels with lower values of yield strength, because of a greater portion of elastic deformation in total deformation [7]. These higher strength steels are usually used for structural parts where high-strength characteristics are desirable, such as in car body design, for A and B pillars, front rails, and reinforcement beams in doors and roofs [8,9,10].

One such steel that is often used for the production of structural parts of vehicles is the dual-phase steel “DP”. These steels have higher strength characteristics, good plasticity, and weldability. Their tensile strength ranges from 500 to 1200 MPa, and total elongation ranges from 11 to 23% [11,12]. Bake hardening and work hardening can further increase the strength of this type of steel. A soft ferrite matrix with islands of harder martensite can be observed during microstructure analysis of the DP steel [13]. The amount of martensite in DP steels ranges from 3 to 30 vol.%. Phase transformation of austenite to martensite has the main influence on the final mechanical properties of dual-phase steel [14,15].

The hat-shaped sheet metal parts are often used for springback investigation and evaluation in research papers [16,17,18]. They are applied for verification of the springback numerical predictions. The hat-shaped part was used by Yoshida-Uemori [17] for verification of their proposed hardening model which can describe the transient Bauschinger effect and workhardening stagnation which appears at reverse loading. The implementation of the Bauschinger effect and workhardening stagnation can improve springback prediction [18,19,20]. This shape is also present in reinforcement beams of doors and roofs of passenger cars made of materials with high values of yield and tensile strength. Koncyak et al. [16] studied the impact of blank holding force on the springback prediction of a hat-shaped part made of AHSS steel. They found out that the Hill48 yield criterion was more accurate in predicting springback when a higher value of blank holding force was applied during forming in comparison with the Yoshida yield criterion. The Lawanwong et al. [17] research aimed to investigate an innovative process called “double-action bending” to eliminate the springback of the hat-shaped part made of DP steel. The work of Uemori et al. [18] was aimed to study the influence of the Bauschinger effect and anisotropy on springback prediction of U and hat-shaped parts made of aluminum alloys. They found that the right combination of anisotropic yield function and isotropic-kinematic hardening model is crucial for accurate springback prediction. Mulidran et al. [19] performed an experimental and numerical investigation of the springback of a hat-shaped part made of DP steel. Their work was focused on the accurate evaluation of springback prediction when using different types of material models in two types of tool design.

Numerical simulations of forming processes are usually based on the finite element method (FEM). Correct inputs and parameters must be used in numerical simulation to obtain relevant results. In metal forming simulations four types of parameters exist that are vital for simulation accuracy [20,21,22]. These categories include tool design, forming process, material, and numerical parameters [19]. The influence of these parameters was studied by numerous researchers. Tomas et al. [23] performed a FE (Finite element) simulation of drawing a rectangular-shaped part to verify the experimental drawing results. They used Hill48 and Hill90 yield criteria in combination with Krupkowski and Hollomon hardening laws. Their results show that prediction with Hill48 yield criterion in combination with Krupkowski hardening law showed very low deviation from the experimental thickness values. Mulidran et al.’s study was aimed at the springback prediction A-pillar part made of light-weight alloy. The simulations were performed using different types of yield criteria in combination with the Voce hardening law in forming the simulation. Springback evaluation was conducted in three planes, and the simulation outcomes were compared with the experimental ones [24]. Solfronk et al. [25] studied the effect of various numerical parameters on the springback prediction of sandwich material. Their results suggest that the use of volume elements does not significantly improve springback prediction, but computational time increases greatly.

The friction has a significant impact on the formability of steel sheets. The reduction of friction has a positive impact on the formability of metal sheets in general [26,27,28]. The work of Evin and Tomas [26] aimed to study the effects of Fe content in the Zn-Fe coating of steel sheets on friction during forming. They tested the formability of steels in the dry condition, with Anticorit lubricant and microtene foil as a lubricant. Their results suggest that the Fe content of the Zn-Fe coating should range from 7% to 12%. Srinivasan et al. [29] studied the effects of friction on the springback of steel sheets in air bending. Their results suggest that a decrease in friction increases the springback and reduces the bending force.

The presented article deals with the springback evaluation of a hat-shaped part made of dual-phase steel when two different process parameters change—friction and blank holding force. The effect of various types of yield criteria and hardening laws on springback prediction accuracy was evaluated. The numerical springback results obtained by the numerical simulation were compared with the experimental ones. The reason for conducting this research was to study the effect of PE foil on the springback, which is not commonly used as lubricant in forming, and also four types of variable blank holding forces were tested. The novelty of the presented article is the comprehensive analysis of the impact of blank holding force and friction on the springback of a hat-shaped part made of HCT600X+Z steel. Moreover, the influence of the material model on the accuracy of the springback analysis affected by friction and BHF is investigated.

## 2. Materials and Methods

### 2.1. Material

**Figure 1:**
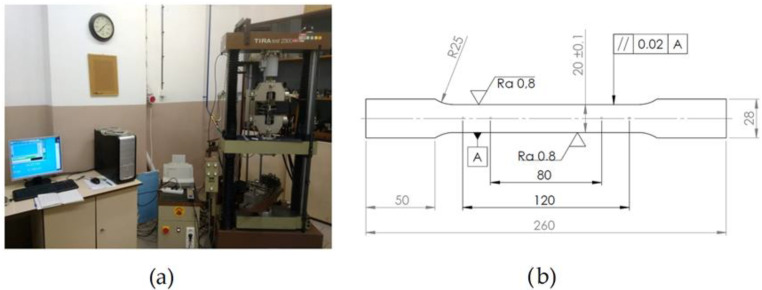
Hydraulic testing machine TIRAtest2300 (**a**); tensile test specimen (**b**).

Dual-phase steel HCT600X+Z with zinc coating was used in the experimental work; the thickness of the steel was 0.80 mm. The first types of DP steels have been developed in the 1970s [15]. The high strain hardening rate of dual-phase steel can be attributed to its microstructure. Different steels, hence, behave differently and have specific properties during plastic deformation because of their microstructure [30,31,32,33]. It is important to know the properties and characteristics of the material, especially when performing a numerical simulation of forming processes [34,35]. The uniaxial tensile tests on standardized specimens were performed to obtain mechanical and plastic properties for the tested material. The obtained results from uniaxial tests are shown in Table 1. The hydraulic testing machine TIRAtest2300 and tensile test sample are depicted in Figure 1.

Five tensile test samples were produced and tested in 0°, 45°, and 90° in the rolling direction to measure the properties. The elongation and width of samples were measured by the extensometers.

### 2.2. Experimental Setup

**Figure 2:**
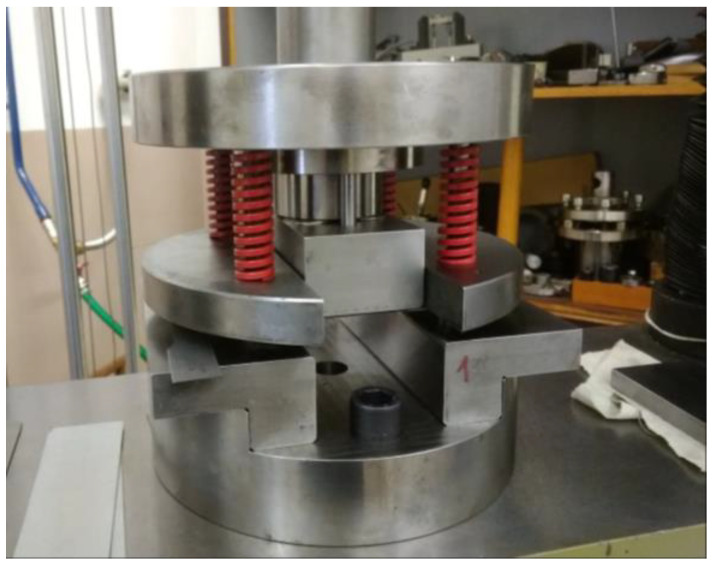
The bending tool designed for springback investigation is positioned in the TIRAtest2300 machine.

To study springback, the influence of the process, and design parameters, a bending tool was designed to produce hat-shaped stampings (Figure 2). This instrument enables changes in tool design and forming process parameters. Mainly punch and die radius, punch width, punch stroke, and blank holding force can be changed. The forming tool can be used in the TIRAtest2300 machine, thus punch stroke and forming force can be measured during the testing procedure.

**Figure 3:**
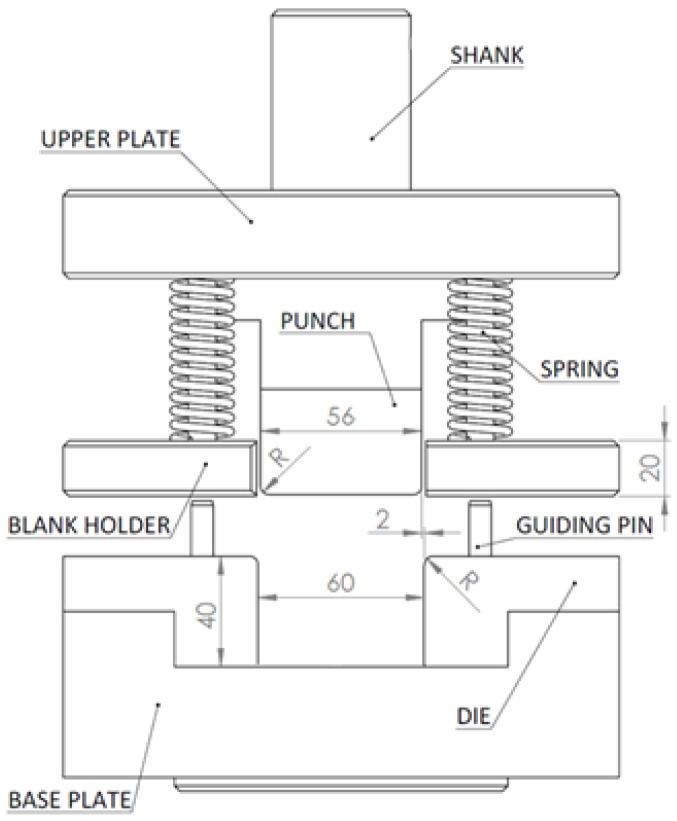
Scheme of bending tool used for springback investigation with dimensions (mm).

Figure 3 shows the main dimensions (mm) of the bending tool used in the experiment. The punch had 5 mm radius and the die had 7 mm radius. The die clearance c_d_ was set to 2 mm. The bending speed v_b_ was 20 mm per minute. Forming depth s_f_ was 17 mm. The blank holder force was applied by four springs, two springs for each side of the blank. Four types of springs were used in the experiment; each one had a different value of the maximum force of one spring F_BHM_ at the end of the punch stroke and the initial force of one spring F_BHI_ (initial contact of punch with blank). The external diameter of the spring was 20 mm and the length of the spring was 64 mm. Force values for springs are shown in Table 2.

**Figure 4:**
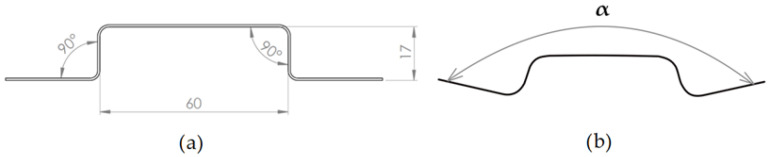
Hat-shaped part with ideal geometry and dimensions (mm) (**a**); scheme of measuring angle α (°) (**b**).

The blank had a rectangular shape and its dimensions were 40 × 150 mm. To study the effect of friction, one-half of the samples were produced under dry conditions without lubrication and the other half of the stampings were produced using PE foil with a thickness of 0.09 mm. The springback was investigated on the hat-shaped part. To examine the springback of the hat-shaped part, the arms angle after springback α [°] was measured. Springback angle was measured on 5 samples for each type of parameter combination regarding blank holding force and friction in the bending process. The photographic record of the produced sample was imported into CAD software and angle α was measured with the sketching tools in the CAD program. The ideal stamping and scheme of measuring of α is shown in Figure 4.

### 2.3. Simulation Setup

**Figure 5:**
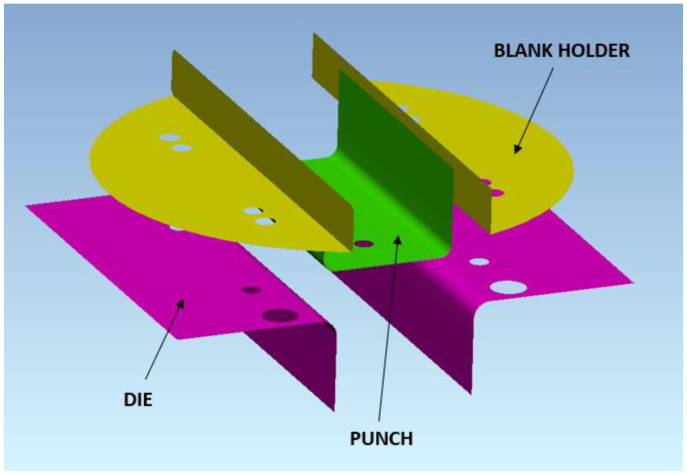
Bending tool in the PAM-STAMP software environment.

Bending and springback simulation was performed using PAM-STAMP 2G software (ESI Group, Rengis, France). Mentioned software is widely used for sheet-metal forming simulations and uses an explicit solver. The CAD model of the bending tool (Figure 5) was imported into the software, and blank dimensions and its position were defined. The blank was mashed with shell elements, the initial size of the element was 2.7 mm, and after refinement, the size of the element was 1.35 mm. The material properties of dual-phase steel were defined using three different yield criteria (Yoshida, Hill48, and Barlat2000) and three different hardening laws (Yoshida-Uemori, Krupkowski, and Hollomon). The blank holding forces values in the simulation were the same as in the experiment, these forces were represented by linear function based on the experimental measuring of spring forces. To represent bending without lubrication, the coefficient of friction value *f* was set to 0.27. Bending simulations with a coefficient of friction value of 0.10 were conducted to represent bending using PE foil. The simulations were designed to analyze the changes in predicted springback caused using different material models, different values of blank holding forces, and friction. Numerical springback results were compared with experimental ones.

#### 2.3.1. Yield Criteria

Cold-rolled steel sheets have different plastic properties in the rolling, the transverse, and the thickness directions. This difference in properties is caused by the manufacturing process. In addition, during forming, deformation-induced anisotropy is present because of microstructure evolution and changes. To perform valid, accurate numerical simulations of forming processes it is important to model the yielding of the material accurately [36,37,38].

**Figure 6:**
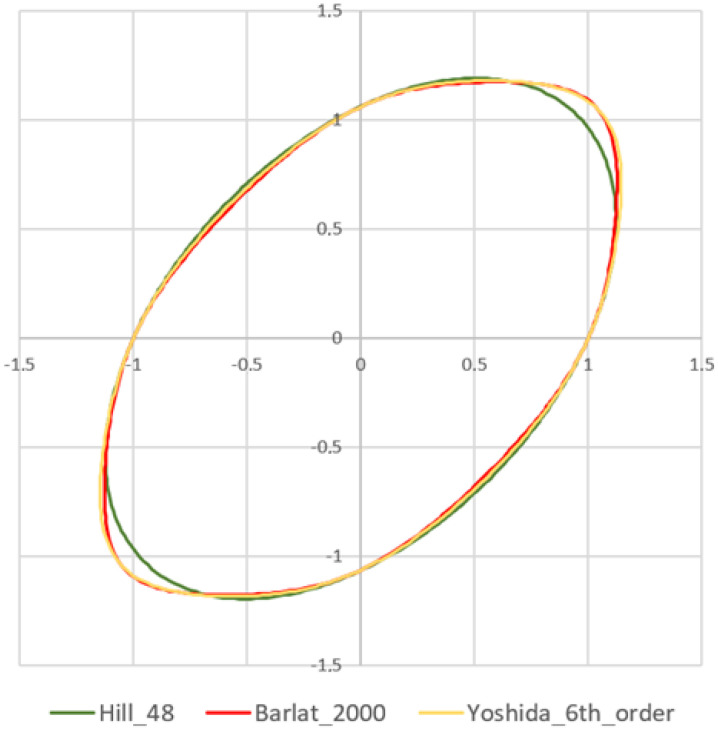
Comparison of yield criteria used in the simulation.

Three yield criteria were used in the springback prediction research. The Yoshida [39], Hill48 [40], and Barlat2000 [41] criteria were utilized in the numerical simulation to evaluate the springback prediction accuracy (Figure 6). The parameters to define the above-mentioned yield criteria are specified in Table 3.

#### 2.3.2. Hardening Rules

Hardening rules, sometimes known as hardening laws, describe how the yield surface changes during the plastic deformation of material [42]. Three main types of hardening rules are used to describe the hardening material of metallic materials in forming simulations. Isotropic hardening rules describe yield surface expansion when stress is applied to the material, but the shape of the yield surface does not change. Kinematic hardening rules describe yield surface translation in stress space; the shape of the yield surface does not change for this type of rule. The last, so-called combined hardening rule uses a combination of isotropic and kinematic hardening to define the behavior of the material under plastic strain [34,42].

To evaluate the influence of hardening rules on the accuracy of springback prediction, three types of rules were used. Two isotropic hardening rules Krupkowski and Hollomon and one combined hardening rule Yoshida-Uemori were used in forming simulations. Parameters for the mentioned hardening rules are shown in Table 4 and Table 5. Isotropic hardening rules are defined as:

- Hollomon

(1) $$\sigma =K\cdot \varphi^{n}$$

- Krupkowski

(2) $$\sigma =K\cdot {\left(\varphi_{0}+\varphi_{pl}\right)}^{n}$$

where *σ* defines the true stress, *K* is the strength coefficient, *n* represents the strain-hardening exponent, *φ*_0_ is the pre-strain, and *φ_pl_* defines the plastic strain.

The Yoshida-Uemori [43] hardening rule can describe the Bauschinger effect and stagnation of workhardening in reverse loading, which is especially important for the springback prediction.

## 3. Results

The springback results from the simulations were compared with experimental ones. Angle α [°] measured from experiment samples was compared with the angle from numerical simulations. In addition, the impact of the blank holder force and friction was studied. The differences of the predicted springback angle α from the experimental values are investigated.

### 3.1. Analysis of Springback Results from Experiments

**Figure 7:**
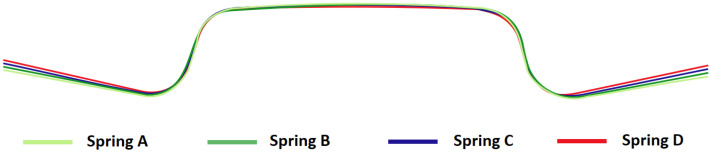
Comparison of hat-shaped parts made using different types of springs and values of blank holding force.

**Figure 8:**
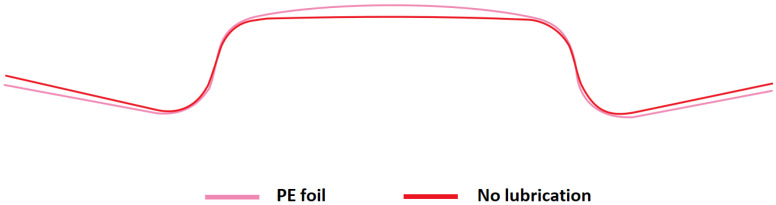
Comparison of hat-shaped parts made with PE foil and no lubrication using D-type springs.

**Figure 9:**
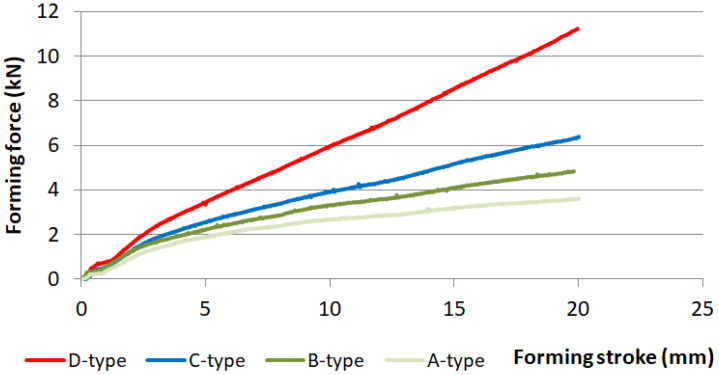
Comparison of forming forces when different springs are used for forming with no lubrication.

The effect of blank holding force and friction on the springback of the hat-shaped part was investigated. Four types of springs were used in the experiment. The impact of friction was studied using samples without lubrication and samples wrapped in PE foil. The shape comparison of samples produced while using four different types of springs is shown in Figure 7. In Figure 8, the shape comparison of two samples produced using PE foil and no lubrication, while using springs of type D, is presented. The comparison of forces during forming with different types of springs and no lubrication is shown in Figure 9. The values of angle α for samples made using different types of springs without lubrication are presented in Table 6, and angle α values for parts produced with lubrication are shown in Table 7.

**Figure 10:**
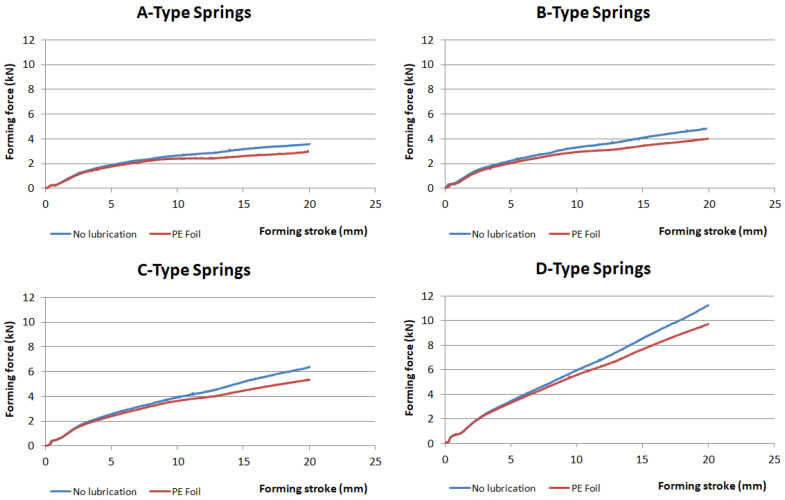
Forming force comparison when different types of springs are used in the experiment with and without lubrication.

Figure 10 shows a comparison of forming forces for each type of spring with and without lubrication.

After profile comparison of stamped parts produced with four types of springs, one can say that the parts made with lower values of blank holding force show less springback of the part in comparison with a part made with higher blank holding force values. The increase of springback when higher BHF is used can be attributed to the stress which is also higher, thus increasing the impact of elastic deformation on the stamped part. Angle α measured after springback was increased by 2.5° when a part was produced with the weakest springs A-type springs, thus reducing the springback effect. The decrease in blank holding force also affects the forming force causing it to decrease up to three times. PE foil used as a lubricant in the experiment can lower forming force F_F_ by up to 20%. The use of PE foil can reduce the springback effect by up to 8%. Use of lubrication, PE foil, can reduce the stress in the part during forming, which can result in a reduction of elastic deformation on the overall deformation, thus reducing the springback of the part.

### 3.2. Analysis of Springback Prediction Results

To evaluate the effects of yield criteria and hardening rules on springback, predictions forming simulations in PAM-STAMP software were conducted. In these simulations, nine combinations of material models were examined (Table 8).

**Figure 11:**
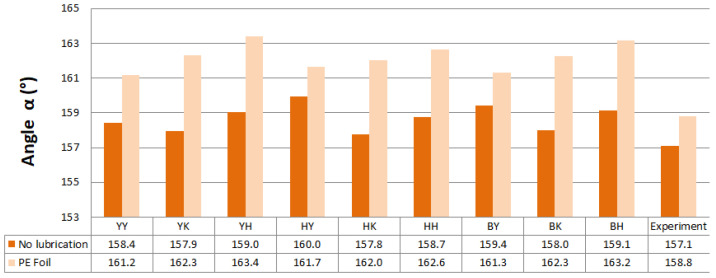
Predicted values of angle α after springback calculation when springs type A were used.

**Figure 12:**
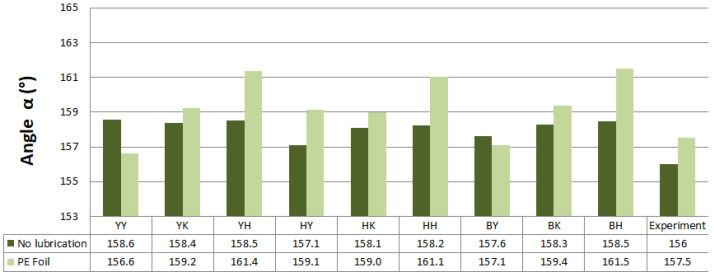
Predicted values of angle α after springback calculation when springs type B were used.

**Figure 13:**
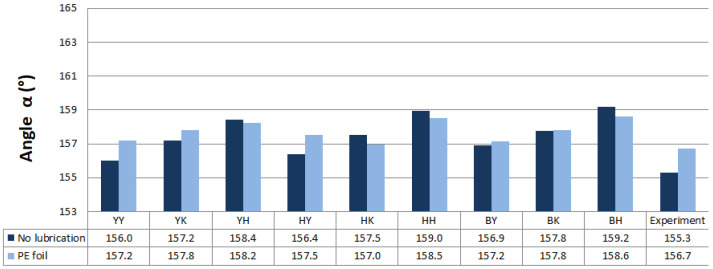
Predicted values of angle α after springback calculation when springs type C were used.

**Figure 14:**
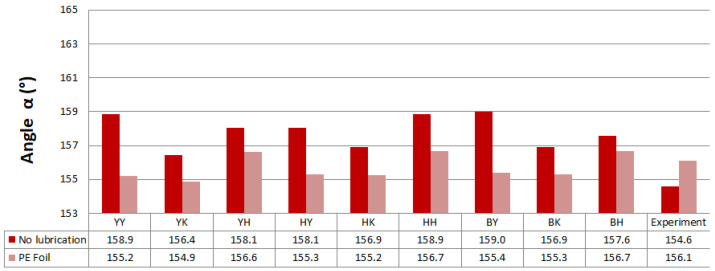
Predicted values of angle α after springback calculation when springs type D were used.

Predicted values of angle α after springback calculation with the use of different combinations of yield criteria and hardening laws for the spring types A and B with and without lubrication are shown in Figure 11 and Figure 12. Predicted values of angle α when spring types C and D were used with and without lubrication are shown in Figure 13 and Figure 14.

Material models with the Yoshida-Uemori hardening law were more accurate in springback prediction based on the numerically predicted results of angle α when a lower value of blank holding force (spring types A and B) was used in most cases. Yield criterion Hill48 in combination with the Krupkowski hardening rule showed the best correlation of numerical results with experimental results of angle α for spring type A when no lubrication was used. When lubrication was used, the Yoshida yield criterion and Yoshida-Uemori hardening law correlated best with the experimental results of angle α. The best correlation with experimental results when blank holding force represented springs type B with and without lubrication was attained using the Barlat2000 yield surface model in combination with the isotropic–kinematic hardening law Yoshida-Uemori.

Predicted angle α for the hat-shaped part made with the use of C-type springs and no lubrication showed the best correlation with the experimentally obtained value when the yield surface model Yoshida in combination with isotropic–kinematic hardening model Yoshida-Uemori was used. When lubrication was present, Hill48, in combination with Hollomon, showed the best correlation with experimental results of angle α. The Yoshida surface model in combination with Yoshida-Uemori showed the best correlation with the experimental value of angle α when D-type springs were represented in simulation and no lubrication was used. When lubrication was represented, the Yoshida yield locus model and Hollmon hardening law showed the best correlation with the experiment.

Overall, springback was underestimated by numerical prediction; higher values of angle α were predicted compared to experimental ones. When D and B-type springs were represented and lubrication was used in the simulation, the springback prediction was overestimated in some cases.

Deviations of predicted springback angle β_SIM_ (°) from the experimental springback values β_EXP_ are shown in Table 9, Table 10, Table 11 and Table 12. Springback angle β (°) can be calculated as:

(3) β=180°−α

Deviations of springback angle β were calculated according to the formula:

(4) $$\mathrm{Dev}\;X=\frac{X_{\mathrm{sim}}-X_{\exp}}{X_{\exp}}\cdot 100\;\left[\%\right]$$

where Dev X is a deviation of predicted springback angle β_SIM_ from the experimental angle β_EXP_, X_sim_ is the predicted springback angle value, and X_exp_ is the experimental springback angle value.

## 4. Discussion

The results presented in this work aimed to evaluate the impact of blank holding force and friction on springback and its prediction. A hat-shaped part made of dual-phase steel was chosen for the study. Recently published research articles on the springback prediction of sheet-metal parts mainly aimed at the investigation of material and process parameters and their impact on the springback prediction in numerical simulations [23,24,42]. Springback prediction and the possible reduction of hat-shaped part springback made of HCT600X+Z steel was the subject of the presented article. The research aimed to study the impact of material models on the accuracy of springback prediction, and the possible springback reduction measures achieved with the change of blank holding force and friction. Other works [36,44,45] were focused on springback reduction with the use of an innovative tool or process design. The work of Lawanwong et al. [17] used a double-action bending tool design, which helped with the springback reduction of the hat-shaped part.

The experimental and numerical results show the importance of blank holding force and friction on the springback effect. Deviations of the angle after springback β from the experimental springback values for all material model combinations were calculated. Arms angle α was reduced when springs with the lowest value of force were used in the experiment and simulation. The above-mentioned angle can be further reduced when PE foil is used as a lubricant during forming operation. The lowest springback was measured when A-type springs were used in combination with PE foil, which helped to reduce friction. Results regarding the impact of blank holding force and friction comply with the works of Saravanan et al. [46] and Damtew [47].

The numerical results show that the springback was underestimated in 65 of 72 predictions, and thus underrated the impact of elastic deformation on the total deformation. Six cases of overestimating springback were observed when the highest values of BHF in combination with a lower friction coefficient were used in numerical predictions. When the highest BHF and lubrication was used in simulation, lower deviations of predicted springback were measured compared to predictions in which dry friction was represented (Table 12). The opposite was observed when the lowest BHF was used. Predictions were more accurate when dry friction was represented compared to springback results when lubrication was applied (Table 9). The Yoshida-Uemori isotropic–kinematic hardening rule demonstrated the best correlation of springback values with the experimentally measured ones in some cases. For example, when the combination of lowest BHF and lubrication was used in simulation (Table 9), the springback predictions showed the lowest deviations from experimental results when the Yoshida-Uemori hardening model was used in combination with all types of yield criteria. However, when dry friction was represented in simulation, the Krupkowski hardening rule in combination with Hill48, Barlat2000, and Yoshida showed the lowest deviations of predicted results from experimental ones. Thus, we can state that friction has impact on the springback predictions. The combined isotropic–kinematic hardening rule achieved more accurate springback predictions only in a limited number of cases based on our results.

The presented results can be applied for dry forming (process without lubrication) and for forming with lubrication (PE foil) when a predominant plane strain is present during forming operation (U-bending, V-bending, hemming, flanging). The smallest deviations of predicted springback values from the experimental ones were achieved for the stamping made with B-type springs when the Barlat yield surface model was used in combination with the Yoshida-Uemori hardening rule. The deviation of the angle β was 1.9%. These results are supported by similar findings published by Baara [48]. Their results show that a combined (isotropic–kinematic) hardening rule showed more accurate springback prediction. These results also comply with the works of Jin [49] and Naofal [50].

## 5. Conclusions

The springback effect and its accurate prediction are one of the challenges that are still present in metal forming processes. In this work, the impact of blank holding force and friction on the springback of a stamped part made of HCT600X+Z steel was studied. To evaluate springback prediction accuracy nine combinations of yield criteria and hardening rules were tested in the numerical simulation software PAM-STAMP 2G. Based on the experimental and numerical results, the following conclusions can be stated:

- Blank holding force has an impact on the springback of the hat-shaped part, when lower values of blank holding force were applied on the blank during forming, then less springback of the part was observed and lower forming force F_F_ was measured. Hat-shaped parts made with the use of the weakest springs experienced a 10% reduction in springback compared to parts produced with the strongest springs.
- The use of PE foil as a lubricant can reduce the springback effect by up to 8%.
- The Yoshida-Uemori hardening rule showed more accurate predictions of springback in a limited number of cases compared to isotropic hardening rules. Accuracy of springback predictions is impacted by friction, BHF, hardening rules, and yield criteria.
- Springback predictions were more accurate for parts made with the lowest values of blank holding force when a higher value of friction coefficient was used. Parts made using the highest values of blank holding force showed more accurate springback prediction when lower friction was applied in simulation.

## Figures and Tables

**Table 1:** The mechanical properties and formability parameters of the HCT600X+Z steel.

Dir. [°]	E [GPa]	R_p0.2_ [MPa]	R_m_ [MPa]	A_80_ [%]	*r* [-]	*r_m_* [-]	*n* [-]	*n_m_* [-]
0		405	656	24.4	0.745		0.197	
45	199	423	661	22.3	0.883	0.862	0.186	0.188
90		430	670	25.5	0.934		0.183	

E—Young’s modulus, R_p0.2_—yield stress, R_m_—ultimate tensile strength, A_80_—total elongation, *r*—plastic strain ratio, *n*—strain hardening exponent, *n_m_*—average value of strain hardening exponent, *r_m_*—average value of plastic strain ratio.

**Table 2:** Force values of system compression springs used in the bending tool for springback investigation.

Spring Type	F_BHI_ (kN)	F_BHM_ (kN)	Maximum Allowed Compression (mm)
A	0.10	0.35	32.0
B	0.15	0.53	25.6
C	0.20	0.80	24.0
D	0.30	1.30	19.2

**Table 3:** Parameters used to define Hill48, Barlat2000, and Yoshida yield criteria.

*r*_0_ (-)	*r*_45_ (-)	*r*_90_ (-)	*σ*_0_ (MPa)	*σ*_45_ (MPa)	*σ*_90_ (MPa)	*σ_biax_* (-)
0.745	0.883	0.934	405	423	430	1.04

*r*_0_—plastic strain ratio in 0° to the rolling direction, *r*_45_—plastic strain ratio in 45° to the rolling direction, *r*_90_—plastic strain ratio in 90° to the rolling direction, *σ*_0_—yield stress in 0° to the rolling direction, *σ*_45_—yield stress in 45° to the rolling direction, *σ*_90_—yield stress in 90° to the rolling direction, *σ_biax_*—biaxial stress.

**Table 4:** Hollomon and Krupkowski models parameters.

Model	*K* (MPa)	*n* (-)	*φ*_0_ (-)
Hollomon	1 041	0.165	-
Krupkowski	1 040	0.168	0.0036

**Table 5:** Yoshida-Uemori model parameters.

Y(MPa)	*a*_0_ (-)	*C*_1_ (-)	*C*_2_ (-)	*b* (-)	*m* (-)	*Rsat* (-)	*h* (-)
405	108.6	115.9	215.24	171.87	8.24	182.30	0.36

The material yield strength is Y, the material parameter *C*_1_ and *C*_2_ control the dynamic hardening rate, the material boundary surface parameter *a*_0_, the material parameters *b* and *m* control the isotropic hardening, *Rsat* is the saturation value, and the material parameter *h* controls the work hardening hysteresis.

**Table 6:** Values of angle α and forming force F_F_ were measured when no lubrication was used in the experiment.

Spring Type	α (°)	F_F_ (kN)
A	157.1	3.60
B	156.0	4.83
C	155.3	6.38
D	154.6	11.25

**Table 7:** Values of angle α and forming force F_F_ were measured when PE foil was used in the experiment.

Spring Type	α (°)	F_F_ (kN)
A	158.8	2.98
B	157.5	4.05
C	156.7	5.34
D	156.1	9.72

**Table 8:** Combinations of material models used in forming simulations.

Abbreviation	Yield Criterion	Hardening Rule	Hardening
YY	Yoshida	Yoshida-Uemori	isotropic-kinematic
YK	Yoshida	Krupkowski	isotropic
YH	Yoshida	Hollomon	isotropic
HY	Hill48	Yoshida-Uemori	isotropic-kinematic
HK	Hill48	Krupkowski	isotropic
HH	Hill48	Hollomon	isotropic
BY	Barlat2000	Yoshida-Uemori	isotropic-kinematic
BK	Barlat2000	Krupkowski	isotropic
BH	Barlat2000	Hollomon	isotropic

**Table 9:** The deviations of predicted springback angle β_SIM_ from the experimental springback value β_EXP_ when springs type A were used.

Lubrication	Parameter	YY	YK	YH	HY	HK	HH	BY	BK	BH	Experiment
no	β (°)	21.6	22.1	21.0	20.1	22.3	21.3	20.6	22.0	20.9	22.9
	Dev β (%)	−5.7	−3.7	−8.4	−12.4	−2.8	−7.2	−10.1	−4.0	−8.9	-
yes	β (°)	18.9	17.7	16.6	18.3	18.0	17.4	18.7	17.7	16.9	21.2
	Dev β (%)	−11.1	−16.7	−21.8	−13.5	−15.2	−18.0	−11.9	−16.4	−20.5	-

**Table 10:** The deviations of predicted springback angle β_SIM_ from the experimental springback value β_EXP_ when springs type B were used.

Lubrication	Parameter	YY	YK	YH	HY	HK	HH	BY	BK	BH	Experiment
no	β (°)	21.4	21.6	21.5	22.9	21.9	21.8	22.4	21.7	21.5	24.0
	Dev β (%)	−10.7	−9.9	−10.6	−4.6	−8.8	−9.3	−6.8	−9.6	−10.3	-
yes	β (°)	23.4	20.8	18.7	20.9	21.0	19.0	22.9	20.7	18.5	22.5
	Dev β (%)	4.0	−7.6	−17.1	−7.3	−6.7	−15.8	1.9	−8.2	−17.7	-

**Table 11:** The deviations of predicted springback angle β_SIM_ from the experimental springback value β_EXP_ when springs type C were used.

Lubrication	Parameter	YY	YK	YH	HY	HK	HH	BY	BK	BH	Experiment
no	β (°)	24.0	22.8	21.6	23.6	22.5	21.0	23.1	22.2	20.8	24.7
	Dev β (%)	−2.8	−7.7	−12.7	−4.5	−9.0	−14.8	−6.5	−10.0	−15.7	-
yes	β (°)	22.8	22.2	21.8	22.5	22.9	21.5	22.9	22.2	21.4	23.3
	Dev β (%)	−2.0	−4.7	−6.5	−3.5	−1.9	−7.7	−1.9	−4.7	−8.2	-

**Table 12:** The deviations of predicted springback angle β_SIM_ from the experimental springback value β_EXP_ when springs type D were used.

Lubrication	Parameter	YU	YK	YH	HY	HK	HH	BY	BK	BH	Experiment
no	β (°)	21.1	23.6	21.9	22.0	23.1	21.2	21.0	23.1	22.4	25.4
	Dev β (%)	−16.8	−7.2	−13.6	−13.6	−9.0	−16.7	−17.3	−9.0	−11.7	-
yes	β (°)	24.8	25.1	23.4	24.7	24.8	23.3	24.6	24.7	23.3	23.9
	Dev β (%)	3.8	5.1	−2.3	3.4	3.6	−2.3	3.1	3.3	−2.4	-

## Data Availability

Data available on request.
